# The phospholipid membrane compositions of bacterial cells, cancer cell lines and biological samples from cancer patients†

**DOI:** 10.1039/d1sc03597e

**Published:** 2021-09-28

**Authors:** Kira L. F. Hilton, Chandni Manwani, Jessica E. Boles, Lisa J. White, Sena Ozturk, Michelle D. Garrett, Jennifer R. Hiscock

**Affiliations:** School of Physical Sciences, University of Kent Canterbury Kent CT2 7NH UK J.R.Hiscock@Kent.ac.uk; School of Biosciences, University of Kent Canterbury Kent CT2 7NJ UK M.D.Garrett@Kent.ac.uk

## Abstract

While cancer now impacts the health and well-being of more of the human population than ever before, the exponential rise in antimicrobial resistant (AMR) bacterial infections means AMR is predicted to become one of the greatest future threats to human health. It is therefore vital that novel therapeutic strategies are developed that can be used in the treatment of both cancer and AMR infections. Whether the target of a therapeutic agent be inside the cell or in the cell membrane, it must either interact with or cross this phospholipid barrier to elicit the desired cellular effect. Here we summarise findings from published research into the phospholipid membrane composition of bacterial and cancer cell lines and biological samples from cancer patients. These data not only highlight key differences in the membrane composition of these biological samples, but also the methods used to elucidate and report the results of this analogous research between the microbial and cancer fields.

## Abstract 2

This review acts as a repository and comparison of cell membrane phospholipid composition data collected from microbial and cancer fields.

## Graphical abstract

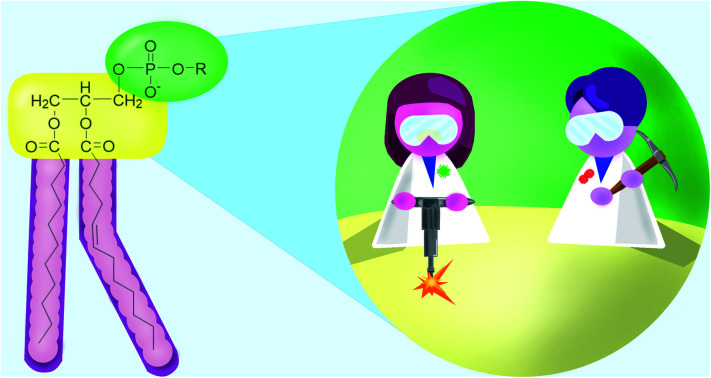

## Introduction

Cancer remains one of the most significant threats to human health, resulting in around 10 million deaths worldwide in 2020.^1^ However, it has been predicted that by the year 2050, an alarming rise in the rates of antimicrobial resistant (AMR) bacterial infections will result in approximately 10 million deaths globally per year, matching the number of deaths currently caused by cancer.^2^

Cancer is caused by gene mutations that can progressively drive the transformation of normal human cells into a malignant form.^3^ Being able to therapeutically target the differences between cancer and normal cells is therefore key for the effective treatment of this disease.^4^ However, many currently marketed cancer drugs exhibit high levels of toxicity towards normal cells, due to the biological similarity that remains between them and their cancerous counterparts.^5^ This means that the therapeutic efficacy of these agents can be limited by deleterious side effects, including neuro- and cardio-toxicity.^6,7^ Intrinsic and acquired resistance to these therapeutic agents is also an issue.^8^

While bacteria possess a far greater degree of structural-diversity compared to healthy eukaryotic cells, making them easier to selectively target, AMR has given rise to bacteria that are resistant to all currently marketed antimicrobial agents.^9^ Moreover, a 34 year hiatus in the identification of novel antibiotics,^10^ primarily caused because of the costs associated with the discovery of new drugs, combined with poor market returns,^9,11,12^ has hampered the development of novel antimicrobial therapeutic agents to meet the growing challenge from AMR infections.

**Fig. 1:**
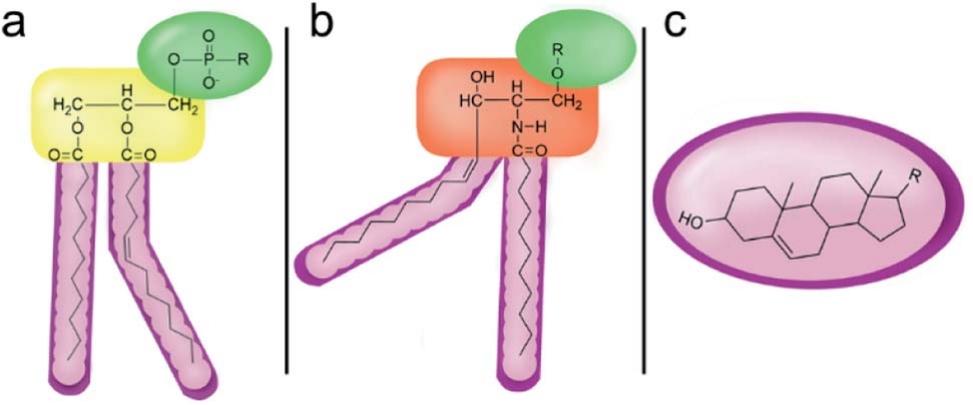
General structure of: (a) glycerophospholipids; (b) sphingolipids and; (c) sterols. The hydrocarbon chain length and the degree of saturation can differ. Pink = hydrophobic residue. Yellow = glycerol linking group. Orange = sphingosine linker. Green = hydrophilic residue.

However, despite the many differences between normal human cells, their cancerous counterparts and bacteria, the cytosol of each is packaged within a cell membrane, that plays essential roles in diverse processes including cellular communication,^13^ energy storage^14^ and structural rigidity.^15^ A major, but more commonly overlooked constituent of this membrane are the lipids. These amphiphilic small molecules, which incorporate a hydrophilic head group and hydrophobic tail group,^16^ can be split into three main classes: glycerophospholipids (commonly referred to as phospholipids), sphingolipids, and sterols (mainly cholesterol in mammals).^17^ The general structures of these lipids are shown in Fig. 1.

Lipid molecules self-assemble, resulting in the formation of a semi-permeable bilayer, in which the polar lipid head groups interact with the aqueous environment, while the hydrophobic tail groups are concealed within the structures core. These bilayers allow passive diffusion of small neutral molecules (O_2_, CO_2_) and solvents (H_2_O), but prevent the diffusion of larger molecules (*e.g.* glucose) and ionic species (Na^+^, Cl^−^).^18^ The incorporation of proteins, carbohydrates and other molecular species within these phospholipid bilayers results in the production of the fully functioning cell membrane, that is able to control the composition of the intracellular environment through closely-regulated molecular transport events.^18,19^

Of the three main classes of lipid found in the cell membrane, phospholipids represent the most prevalent and structurally significant. Alterations to the general chemical structure of the phospholipid head and tail groups, gives rise to an array of amphiphilic molecules, each exhibiting different physicochemical properties. A summary of these structural modifications accompanied by common phospholipid nomenclature has been provided for reference in Tables 1 and 2.^20^ It is important to note that fatty acid tails not only vary in length (typically between 14 and 24 carbon atoms),^21^ but also saturation.^21,22^ Furthermore, R group modifications also occur within the phospholipid head group.^20^ This leads to high levels of chemical and conformational diversity. Further compositional diversity within the cell membrane is brought about by variation in the relative proportion of the different lipids present within it.^17^ It is because of the complexity associated with these amphiphilic molecules, combined with the limitations associated with the construction of synthetic systems, such as membrane transition temperatures, availability of phospholipid samples and associated purchase costs, that we have chosen to limit the focus of our discussion within this review to the phospholipid headgroups only. However, further information relating to the alkyl component of the phospholipids discussed can be found within the ESI,† where available.

**Table 1:** Summary of phospholipid structures, the position of the R group is identified in Fig. 1a.^20^ Overall charge = charge of phospholipid under physiological conditions

Lipid	R-group	Overall charge
Phosphatidic acid (PA)	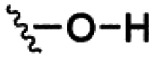	−1
Phosphatidylethanolamine (PE)	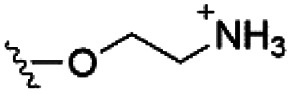	0
Phosphatidylcholine (PC)	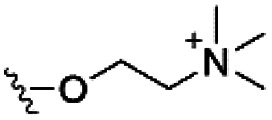	0
Phosphatidylglycerol (PG)	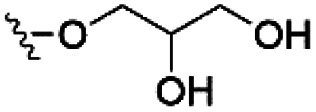	−1
Phosphatidylserine (PS)	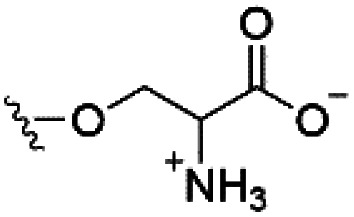	−1
Phosphatidylinositol (PI)	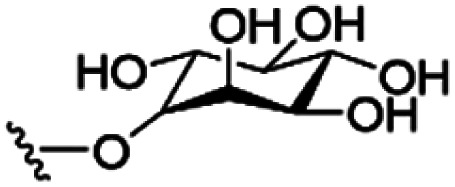	−1
Cardiolipin (CL)	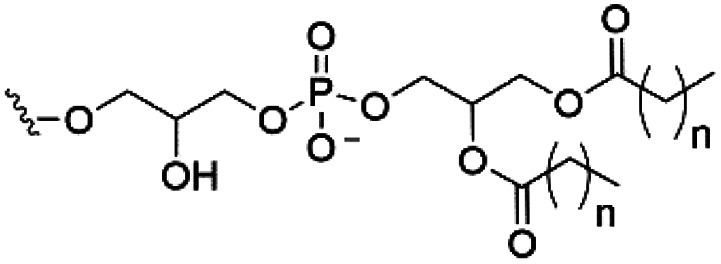	−2
Lysyl-PG	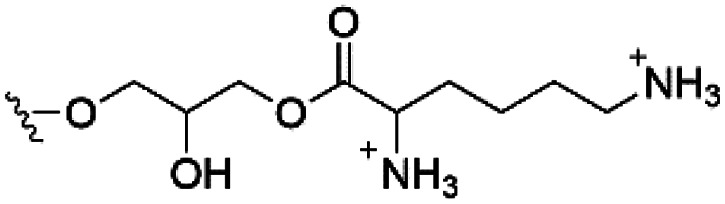	+1

**Table 2:** Other lipids included in this review. The saturation and length of the hydrocarbon chain can differ.^20^ Overall charge = charge of lipid under physiological conditions

Lipid	Structure	Overall charge
Lyso-PA	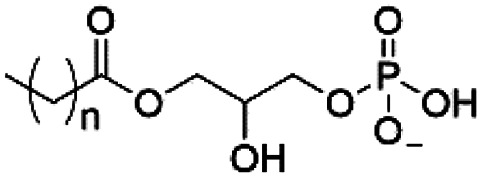	−1
Lyso-PE	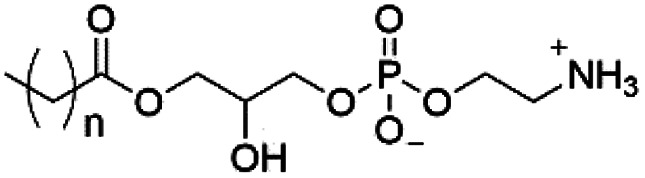	0
Lyso-PC	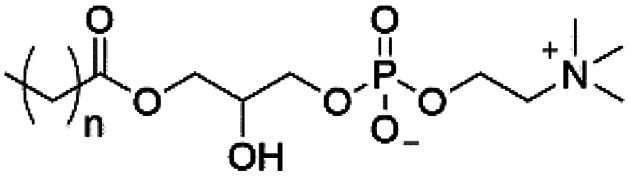	0
Lyso-PI	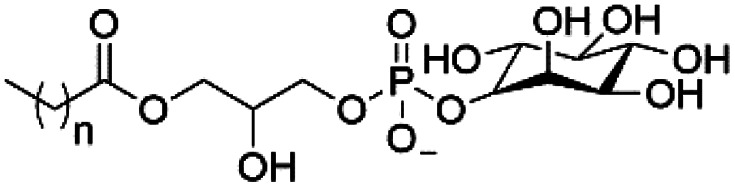	−1
Lyso-CL	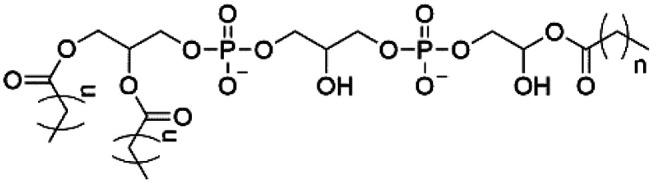	−2
Diacylglycerol (DAG)	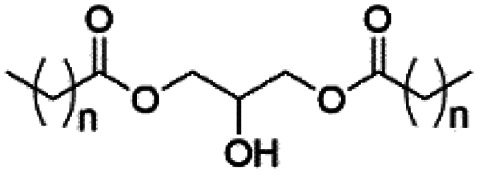	0
Lipid A	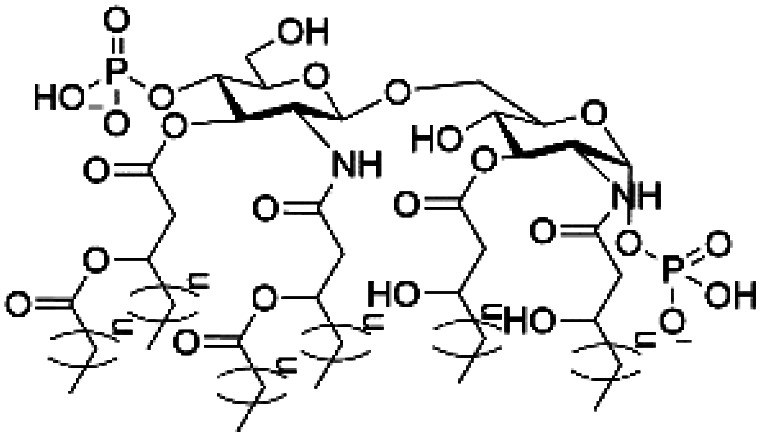	−2
Sphingomyelin (SM)	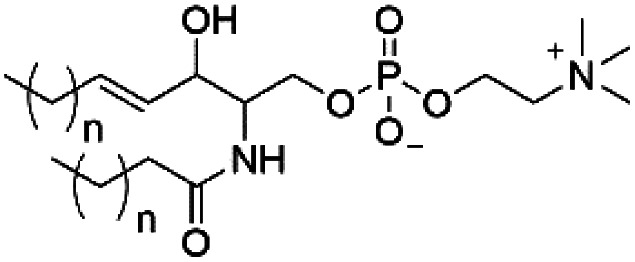	0
Lipoteichoic acid	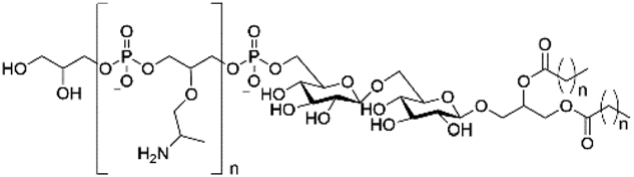	N/A

As important as the phospholipid component of a cell membrane is for cell viability, it can act as a barrier for effective drug delivery,^23–25^ or become the cause of cellular resistance.^26,27^ Alternatively, it can act as a drug target, where cell death is the desired therapeutic outcome.^28–30^ Therefore, understanding interactions between existing or proposed new drugs and phospholipid membranes is of great importance. Examples are emerging of novel membrane-targeting technologies, designed to alter membrane fluidity^31^ or permeability.^32^ Craik and co-workers have designed a novel class of cyclotides identified as potential anticancer therapeutic agents.^33^ Furthermore, O'Shea and co-workers have shown that the anticancer agent, Ophiobolin A (OPA) functions through interaction with specific phospholipids leading to the formation of cytotoxic adducts that cause irreversible lipid bilayer destabilisation.^34^ Gale and co-workers have developed low molecular weight synthetic anion transporter technologies;^35–37^ whilst Jolliffe and co-workers, have recently developed a fluorogenic probe for cell surface PS.^38^

Additionally, there are multiple examples of antimicrobial peptides (AMP) where the mode of action is known to rely upon interaction with, and destabilisation of, bacterial phospholipid membranes.^39^ Finally, within the scope of our own work, we have shown that a novel class of supramolecular self-associating amphiphiles (SSAs) can coordinate to and permeate both bacterial^40^ and cancer cell membranes,^41^ causing SSAs to be identified as anticancer agents,^41^ antimicrobials^40,42,43^ and efficacy enhancers for known therapeutic agents.^41,44^

The analysis herein seeks to aid the effort to understand and characterise interactions between therapeutic candidates and phospholipid membranes by creating a resource detailing the phospholipid membrane composition of cancer and bacterial cells.

## Phospholipid composition of bacterial membranes

Within the scope of this review, we have focused our efforts detailing the phospholipid composition of the ESKAPE pathogens, along with the commonly used models of Gram-negative (*Escherichia coli* – *E. coli*) and Gram-positive (*Bacillus subtilis* – *B. subtilis*) organisms. The ESKAPE pathogens are a group of six nosocomial bacteria (*Enterococcus faecium* – *E. faecium*, *Staphylococcus aureus* – *S. aureus*, *Klebsiella pneumoniae* – *K. pneumoniae*, *Acinetobacter baumannii* – *A. baumannii*, *Pseudomonas aeruginosa* – *P. aeruginosa*, and *Enterobacter* spp.) that the CDC has listed as the ‘biggest’ threats to human health.^45–50^ This is due to the ability of these bacteria to evolve new resistance mechanisms in addition to those currently identified for this group of pathogens.^51–53^

In general, the outer phospholipid membrane of bacterial cells contains mixtures of polar phospholipids such as PE and PG.^54–56^ Therefore, traditionally these bacterial phospholipid membranes have been modelled in synthetic systems using a mixture of PE : PG in a 3 : 1 ratio.^55,57^ However, as detailed in Table 3, this is not representative of many naturally derived bacterial membranes. For example, although Gram-negative *E. coli* has a bacterial phospholipid composition that is very similar to these conventional model systems (PE : PG : CL 75 : 20 : 5),^57^ this is not the case for Gram-positive methicillin-sensitive *S. aureus* U-71 (PG : L-PG : CL 80 : 12 : 5).^58^ Furthermore, the composition of the phospholipid membrane has also been found to be dependent on growth phase^59,60^ and life-cycle,^61,62^ as highlighted with methicillin sensitive *S. aureus* 209P. In log phase growth the phospholipid composition of this bacteria was found to be: PG : L-PG : CL 79 : 14 : 4 however, in stationary phase the phospholipid membrane composition alters to PG : L-PG : CL 66 : 10 : 14.^63^ Additionally, it is well known that phospholipid membrane composition is dependent on bacterial culture conditions. For example, *P. aeruginosa* grown in synthetic cystic fibrosis media (SCFM) showed lower levels of PC compared to *P. aeruginosa* grown in Muller–Hilton broth (MHB).^64^ To more accurately simulate the natural biological environment, the phospholipid composition of the bacterial were also studied in SCFM with added DOPC (SCFM-PC).^65^ As with SCFM only, there was a general increase in comparative quantities of those lipids present within *P. aeruginosa* grown in SCFM-PC, compared to that grown in MHB. This was most notable for PC and PG phospholipids. Interestingly, the minimum inhibitory concentration (MIC) values obtained for a variety of well-known antibiotics (carbenicillin, gentamicin, ciprofloxacin, colistin) against *P. aeruginosa* grown in these different media was also found to alter. With the bacteria grown in MHB showing higher MIC values compared to SCFM.^64^

**Table 3:** Summary of % bacterial phospholipid membrane compositions. Where ‘—’ is shown, the presence of this phospholipid was not confirmed or was shown to be absent within experimental limitations. Where the phospholipid composition does not equal 100%, the remaining phospholipids either could not be identified, or the membrane is also comprised of some less prevalent phospholipids which have been omitted from this summary table. These less common phospholipids include: PA, PC, PS, L-PE, L-CL. For full details, including those relating to alkyl chain composition, culture conditions and phospholipid source, please see Table S1^a^

Bacterial strain	PG	L-PG	DAG	CL	PE	Growth phase	Analysis method
***E. faecium* (Gram +ve)**							
R446 (ref. 66)	15	16	23	47	—	Stationary	MS
S447 (ref. 66)	34	14	13	29	—	Stationary	MS

***S. aureus* (Gram +ve)**							
USA300 (ref. 67)	60	30	4	2	—	Log	MS
USA300 (ref. 67)	62	20	3	4	—	Stationary	MS
DSM 20233 (ref. 68)	50	20	—	1	—	Log	TLC
*fakA* mutant^67^	70	25	3	1	—	Log	MS
*fakA* mutant^67^	60	22	5	6	—	Stationary	MS
CB1118 (ref. 69)	84	12	—	7	—	Unknown	TLC
CB2205 (ref. 69)	84	25	—	7	—	Unknown	TLC
209P^70^	79	14	—	4	—	Log	TLC
209P^70^	66	10	—	5	—	Stationary	TLC
U-71 (ref. 58)	80	12	—	5	—	Unknown	TLC

***K. pneumonia* (Gram −ve)**							
Smooth mutant^71^	5	—	—	6.5	82	Unknown	TLC
005 (ref. 72)	35	—	—	—	59	Unknown	MS

***A. baumannii* (Gram −ve)**							
HO1–N^73^	10	—	—	7	73	Unknown	GC

***P. aeruginosa* (Gram −ve)**							
PAO1 (ref. 74)	27	—	—	—	69	Stationary	MS
B-219 (ref. 75)	41	—	—	—	65	Unknown	MS

***Enterobacter cloacae* (*E. cloacae*) (Gram −ve)**							
S_w1_ (ref. 76)	23	—	—	3.5	73	Stationary	TLC
S_w1_ (ref. 76)	6	—	—	8	77	Log	TLC
012 (ref. 72)	35	—	—	—	65	Unknown	MS
008 (ref. 72)	42	—	—	—	50	Unknown	MS
AZT-R^76^	17	—	—	2	80	Log	TLC
AZT-R^76^	5	—	—	9	76	Stationary	TLC
AMA-R^76^	17	—	—	3	79	Log	TLC
AMA-R^76^	5	—	—	6	82	Stationary	TLC

***B. subtili*s (Gram +ve)**							
I′1a (ref. 77)	70	3	—	4	22	Log	MS
I′1a (ref. 77)	75	2	—	7	17	Stationary	MS
DSM 3257 (ref. 77)	65	2	—	4	35	Log	MS
DSM 3257 (ref. 77)	30	2	—	10	70	Stationary	MS

***E. coli* (Gram −ve)**							
B^78^	20	—	—	5	75	Log	TLC

a MS = mass spectrometry. TLC = thin layer chromatography, GC = gas chromatography.

For these reasons, the culture conditions used have been detailed in the ESI (Table S1†).

As exemplified in Table 3, the phospholipid composition of AMR bacteria differs significantly from those membranes studied from analogous non-resistant bacterial strains. For example, the clinical pair of daptomycin susceptible (S447) and daptomycin resistant (R446) strains of *Enterococcus faecium*^66^ have a phospholipid composition PG : L-PG : CL : DAG 34 : 14 : 29 : 13 and PG : L-PG : CL : DAG 15 : 16 : 47 : 23 respectively.^66^ This was interesting, as there are a number of unique bacterial lipids that are already associated with antimicrobial resistance including lipid A and teichoic acids.^79^ Therefore, when considering the design and delivery of novel therapeutics to treat AMR infections where interaction with, or permeation through the cell membrane is critical, the differences in phospholipid membrane composition should also be taken into consideration.

## Phospholipid composition of cancer cell membranes

Although cancer initiation normally has a genetic trigger, it has been shown that changes in membrane biophysical properties occur when non-malignant cells undergo malignant transformation.^80^ Quantitative changes in all major classes of phospholipids caused by alterations in lipid regulation pathways, have been identified in many types of cancer cell.^81^ For example, upregulation of PA leads to changes in tumour metabolism, such as the activation of kinases involved in intracellular stress signalling pathways,^82^ while elevated levels of PC result in modulation of enzymes involved in metabolic pathways causing higher proliferation rates.^81^ PI is important for a number of signalling pathways involved in cellular processes such as DNA damage, cell differentiation, proliferation, survival and trafficking, with PI5P known to modulate acetylation of p53 and activity of histone deacetylases and acetylases.^83^

PS in the outer leaflet of the cell membrane is an early marker of apoptosis and has also been shown to play a pivotal role in non-apoptotic cell death as changes in its regulation affect necrosis.^84^ Moreover, increased levels of PS are responsible for the weakening of the immune system in response to cancer.^81^ Furthermore, elevated levels of lyso-PA (Table 2) are considered a potential clinical biomarker in ovarian, colorectal^85^ and pancreatic cancer^86,87^ and, are a potential therapeutic target for novel anti-cancer treatments.^88^ PE is present on both the inner and outer leaflet of the plasma membrane and under normal physiological conditions it is more concentrated in the cytosolic region (inner leaflet). However, in cancer cells, this distribution is reversed with PE being more concentrated in the outer leaflet of the plasma membrane.^81^

In addition to those phospholipids detailed within the scope of this review, the levels of plasmalogens (a subclass of cell membrane glycerophospholipids)^89^ have also been linked to cancer, as effectively reviewed by Messias and co-workers.^90^ This review summarises the regulation of plasmalogens in patients suffering from liver, colon, oesophageal, colorectal and pancreatic cancer. Therefore these data have not be reproduced herein, although we do urge the readers of this review to consider this class of lipids within the study of phospholipid cancer cell membranes, the importance of which has been further highlighted by the work of Fernandes and co-workers.^91^

Table 4 summarises the changes in phospholipid composition in cancer *versus* control samples as defined within the table. We note that there are a number of distinct sample types, exemplified by cancer cell lines and biological samples from cancer patients including tissue, plasma and urine. For this review we have focussed on human cancer cell lines and tissues from patients and have omitted other biological sample types such as urine and plasma, which are not representative of cells. We have also only included those data sets that provide a comparison between the cancer sample and a control sample. Two key examples of this are the sampling of adjacent normal tissue when tumour tissue is analysed from a cancer patient, and the use of a non-cancerous cell line (*e.g.* MCF10A for breast cancer) when a cancer cell line is being studied. This is important as it relates to the question of the differences between the normal *versus* cancer cell, which is then translated into the identification of candidate cancer specific biomarkers. Whilst quantitative numerical data on phospholipid content is provided, there is no standardised format in which these data have been reported. It is often summarised in tables that show whether a specific phospholipid is upregulated or downregulated in the cancer *versus* control sample, therefore this is the approach we have taken when summarising these data in Tables 4 and S2.† Within Table 4, an upwards arrow represents upregulation of a specific phospholipid type and a downwards arrow represents downregulation of this phospholipid in the cancer *versus* control sample. Where there are both upwards and downwards arrows present, this reflects the fact there will be multiple forms of a particular phospholipid present in a cell, due to different alkyl chain lengths and their degree of saturation. Some of these maybe upregulated in a cancer cell, whereas others maybe downregulated.

**Table 4:** Summary of cancer cell phospholipid membrane composition. Upwards arrow indicates an upregulation of specific phospholipids, downwards arrow indicates a downregulation of specific phospholipids. Where there are both upwards and downwards arrows present, this reflects the fact there will be multiple forms of a particular phospholipid present in a cell, due to different alkyl chain lengths and their degree of saturation. Some of these maybe upregulated in a cancer cell, whereas others maybe downregulated. Where ‘—’ is shown, a change in phospholipid content was either not observed or not confirmed within experimental limitations. Tissue refers to human tissue obtained from clinical samples, unless otherwise stated. The cell membranes listed here may also contain some less prevalent phospholipids. These data, alongside alkyl chain composition and, the results of studies that restrict analysis to a limited number of phospholipids, are listed in Table S2. These less common phospholipids include: L-PA, L-PC, and L-PI, L-SM. Phospholipid composition was estimated through mass spectrometry (MS)

Sample type	PC	PE	PI	PG	PS	SM	PA	Control
**Lung**								
Tissue^92^	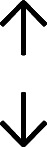	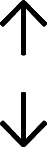	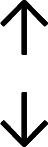	—	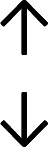	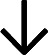	—	Matched distant lung tissue
Tissue^93^	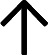	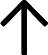	—	—	—	—	—	Distal non-cancerous tissue
Tissue^94^	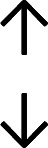	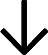	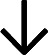	—	—	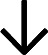	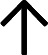	Adjacent normal tissue
Tissue^95^	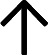	—	—	—	—	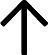	—	Adjacent normal lung tissue
Tissue^96^	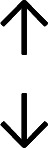	—	—	—	—	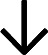	—	Normal lung tissue

**Breast**								
Cell lines^97^	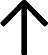	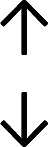	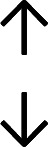	—	—	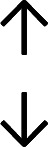	—	MCF10A cell line
Cell lines^97^	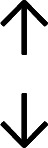	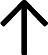	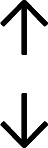	—	—	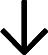	—	MCF10A cell line
Cell lines^97^	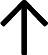	—	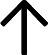	—	—	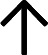	—	MCF10A cell line
Cell lines^98^	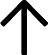	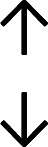	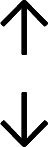	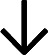	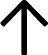	—	—	MCF10A cell line
Cell lines^98^	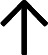	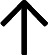	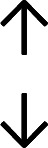	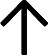	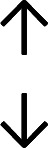	—	—	MCF10A cell line
Tissue^94^	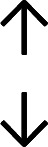	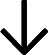	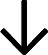	—	—	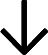	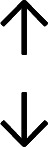	Adjacent normal tissue
Tissue^99^	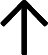	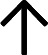	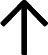	—	—	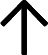	—	Adjacent normal tissue
Tissue^100^	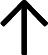	—	—	—	—	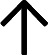	—	Adjacent normal breast tissue
Tissue^101^	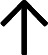	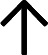	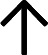	—	—	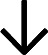	—	Normal breast tissue

**Prostate**								
Cell lines^102^	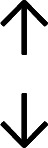	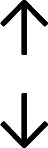	—	—	—	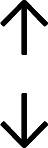	—	PNT1a cell line
Tissue^103^	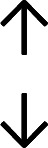	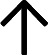	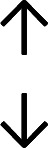	—	—	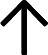	—	Tissue from healthy males

**Colorectal**								
Tissue^104^	—	—	—	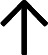	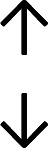	—	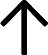	Adjacent normal mucosa
Tissue^94^	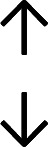	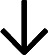	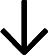	—	—	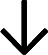	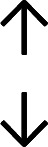	Adjacent normal tissue
Tissue^105^	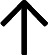	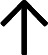	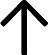	—	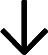	—	—	Adjacent normal mucosa
Tissue^106^	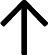	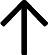	—	—	—	—	—	Adjacent normal mucosa
Tissue^107^	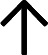	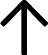	—	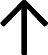	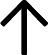	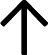	—	Tumour adjacent tissue

**Colorectal liver metastasis**								
Tissue^108^	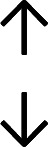	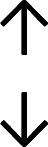	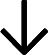	—	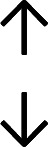	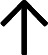	—	Normal liver parenchyma

**Ovarian**								
Tissue^109^	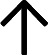	—	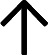	—	—	—	—	Adjacent normal tissue

**Urothelial cancer of the bladder**								
Tissue^110^	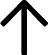	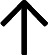	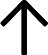	—	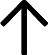	—	—	Benign adjacent tissue

**Gastric**								
Tissue^94^	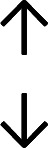	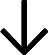	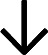	—	—	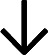	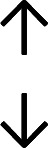	Adjacent normal tissue

**Oesophageal**								
Tissue^94^	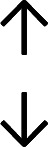	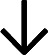	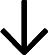	—	—	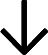	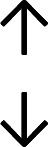	Adjacent normal tissue
Tissue^111^	—	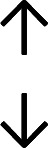	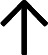	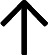	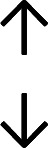	—	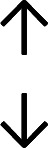	Matched healthy oesophageal epithelium

**Thyroid**								
Tissue^94^	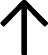	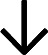	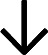	—	—	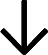	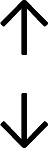	Adjacent normal tissue

**Hepatocellular**								
Tissue^112^	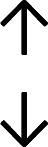	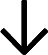	—	—	—	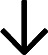	—	Benign tissue

From the data summarised in Table 4, we observe a general trend in which more phospholipids are upregulated in the cancer *versus* the control sample, than downregulated. PC is generally upregulated in both breast cancer cell lines and tissue while the regulation of the remaining phospholipids present appears to be sample specific. A key limitation is the number of reports available for a particular cancer type, which for some such as bladder cancer, is limited to just one. Finally, a key factor in cancer treatment failure is drug resistance. Due to data limitations, it is currently not possible to comment on the differences between the phospholipid content of drug sensitive *versus* resistant cancer cells/samples.

## Conclusions

An increased understanding of cell membrane phospholipid composition in cancer and bacterial cells can provide opportunities to develop novel treatment strategies for cancer and AMR, and thereby begin to countermeasure shortfalls of current therapies. It is the intention that this review of current knowledge into the membrane composition of cancer and AMR cells and, cancerous tissue from cancer patients will aid those wishing to develop novel membrane transport technologies, drug delivery technologies, drug adjuvant technologies, therapeutics, or repurpose approved therapeutics against these diseases. While it is clear that a far greater library of such data would aid current research in these areas, it is also apparent that phospholipid composition studies in the microbiology and cancer fields are undertaken on a different basis. Specifically, we believe that the inclusion of percentage phospholipid composition data, as well as studies into the differences between the phospholipid content of drug sensitive *versus* resistant cancer cells/samples will greatly enhance the applicability of these data.

## Data availability

The datasets supporting this article have been uploaded as part of the ESI† and article references.

## Author contributions

Kira L. F. Hilton: investigation; validation; writing – original draft. Chandni Manwani: investigation; validation; writing – original draft. Jessica E. Boles: investigation; writing – original draft. Lisa J. White: supervision; writing – original draft. Sena Ozturk: writing – original draft. Michelle D. Garrett: funding acquisition; project administration; supervision; writing – review & editing. Jennifer R. Hiscock: conceptualization; funding acquisition; project administration; supervision; writing – original draft, review & editing.

## Conflicts of interest

There are no conflicts to declare.

## Supplementary Material

SC-012-D1SC03597E-s001
