# Supplementary material for: The phospholipid membrane compositions of bacterial cells, cancer cell lines and biological samples from cancer patients
Source: Chem Sci. 2021 Sep 28;12(40):13273–82. doi: 10.1039/d1sc03597e (PMC8529332; doi:10.1039/d1sc03597e)
Supplement: SC-012-D1SC03597E-s001 [file SC-012-D1SC03597E-s001.pdf]

Electronic Supplementary Material (ESI) for Chemical Science.
This journal is © The Royal Society of Chemistry 2021

Comparative phospholipid membrane compositions of bacterial and cancer cell lines

Kira L. F. Hilton,[‡a] Chandni Manwani,[‡a/b] Jessica E. Boles,[a] Lisa J. White,[a] Sena Ozturk,[a] Michelle D. Garrett*[b] and Jennifer R. Hiscock*[a]

Contents

Tables of data 2
Abbreviations 14
References 16

Tables of data

Table S1 – Phospholipid membrane composition of bacterial cell lines. Inner refers to the inner leaflet of the cell membrane, whereat outer refers to the outer leaflet of the cell membrane. Herein, CM = cytoplasmic membrane and OM = outer membrane.

	Phospholipid	Phase of growth (%) Unknown	Log	Stationary	Analysis Method	Growth Media	Specific Notes
***Enterococcus faecium* (Gram +ve)**							
S447[1]	L-PG inner			8.12	2D TLC, LC/ESI-MS/MS	BHI broth	Sat - 14:0 (8 %), 16:0 (26 %), 18:0 (5 %)
(Daptomycin	L-PG outer			6			Unsat - 16:1,7c (13 %), 18:1,6,9c (6 %), 18:1,9c (4 %), 18:1,7c (34 %)
sensitive)	PG whole cell			34			Cyclic - 19:0 (3 %)
	CL whole cell			29			
	DAG whole cell			13			
R446[1]	L-PG inner			7	2D TLC, LC/ESI-MS/MS	BHI broth	Sat - 14:0 (9 %), 16:0 (26 %), 18:0 (5 %
(Daptomycin	L-PG outer			9			Unsat - 16:1,7c (14 %), 18:1,6,9c (6 %), 18:1,9c (3 %), 18:1,7c (29 %)
resistant)	PG whole cell			15			Cyclic - 19:0 (7 %)
	CL whole cell			47			
	DAG whole cell			23			
***Staphylyococcus auerus* (methicillin resistant) (Gram +ve)**							
DSM 20233[2]	PG whole cell		50		TLC	Difco, antibiotic medium III	Lipoteichoic acid was isolated, but quantitative results were not provided.
	L-PG whole cell		20				Information relating to alkyl chain composition not provided.
	CL whole cell		1				
USA300[3]	PG whole cell		60	62	ESI-MS/MS	TSB	PC – 30:0 (8 %), 31:0 (9 %), 32.0 (27 %), 33.0 (25 %), 34:0 (11 %), 35:0 (20 %), 36:0 (2 %)
	L-PG whole cell		30	20			L-PG – 30:0 (6 %), 31:0 (5 %), 32:0 (28 %), 33:0 (27 %), 34:0 (18 %), 35:0 (21 %), 36:0 (2 %), 37:0 (1%), 38:1 (2 %)
	CL whole cell		2	4			
	DAG whole cell		4	3			
*fak*A mutant[3]	PG whole cell		70	60	ESI-MS/MS	TSB	PC - 30:0 (4 %), 31:0 (5 %), 32.0 (20 %), 33.0 (21 %), 34:0 (16 %), 35:0 (32 %), 36:0 (4 %)
	L-PG whole cell		25	22			L-PG – 30:0 (4 %), 31:0 (3 %), 32:0 (19 %), 33:0 (20 %), 34:0 (15 %), 35:0 (21 %), 36:0 (3 %), 37:0 (2%), 38:1 (2 %)
	CL whole cell		1	6			
	DAG whole cell		3	5			
CB1118	PG whole cell	84			2D TLC, spectrophotometry	MHA	Parental strain to produce daptomcyin-nonsusceptibility (resistance)
(USA400)[4]	L-PG inner	11					Information relating to alkyl chain composition not provided.
	L-PG outer	1					
	CL whole cell	5					
CB2205 (20-day	PG whole cell	84			2D TLC, spectrophotometry	MHA	CB2201 – day 1 (O-LPG 11 %, I-LPG 1 %, PG 86 %, CL 4 %)
DAP-	L-PG inner	19					CB2202 – day 5 (O-LPG 12 %, I-LPG 2 %, PG 81 %, CL 9 %)
nonsusceptibilty)[4]	L-PG outer	6					CB2203 – day 14 (O-LPG 15 %, I-LPG 3 %, PG 73 %, CL 13 %)
	CL whole cell	7					Information relating to alkyl chain composition not provided.

***Staphylyococcus auerus* (methicillin sensitive) (Gram +ve)**							
209P[5]	PG whole cell<br>L-PG whole cell<br>CL whole cell	76.5<br>16<br>7			TLC, radiolabelling	Nutrient broth	Information relating to alkyl chain composition not provided.
209P[6]	PG whole cell<br>L-PG whole cell<br>CL whole cell		79.3<br>14.3<br>3.6	65.9<br>10<br>13.7	TLC, radiolabelling	Synthetic broth 1	CL – 14:0 (22.2 %), 15:0 (24.6 %), 16:0 (12.1 %), 17:0 (15.6 %), 18:0 (6.8 %), 19:0 (4.7 %), 20:0 (1.3 %), 22:0 (12.3 %)<br>PG – 14:0 (1.2 %), 15:0 (51.0 %), 16:0 (4.8 %), 17:0 (23.2 %), 18:0 (3.2 %), 19:0 (13.9 %), 20:0 (2.9 %)<br>L-PG – 14:0 (4.7 %), 15:0 (37.0 %), 16:0 (4.1 %), 17:0 (25.0 %), 18:0 (4.9 %), 19:0 (13.5 %), 20:0 (7.1 %)
U-71[7]	PG whole cell<br>L-PG whole cell<br>CL whole cell	80<br>12<br>5			TLC, radiolabelling	Synthetic media 2	Information relating to alkyl chain composition not provided.
***Klebsiella pneumonia* (Gram -ve)**							
Smooth mutant[8]	PE whole cell<br>PG whole cell<br>PA whole cell<br>CL whole cell	82<br>4.5<br>5<br>6.5			TLC, radiolabelling	Synthetic media 3	Information relating to alkyl chain composition not provided.
005[9]	PG whole cell<br>PE whole cell	35<br>59			FAB-MS	Nutrient broth	PG – 27:3 (3.7 %), 28:3 (1.4 %), 29:4 (0.4 %), 29:3 (2.7 %), 29:2 (0.3 %), 30:2 (5.2 %), 31:3 (4.8 %), 31:2 (4.3 %), 32:4 (4.2 %), 32:3 (4.1 %), 32:2 (3.9 %), 34:3 (5.9 %)<br>PE – 30:1 (2.4 %), 31:1 (4.2 %), 32:2 (4.6 %), 32:1 (15.9 %), 32:0 (3.6 %), 33:1 (6.8 %), 34:2 (9.4 %), 34:1 (1.3 %), 34:0 (5.3 %), 35:2 (0.7 %), 35:1 (4.5 %), 36:1 (0.3 %)
***Acinetobacter baumannii* (Gram -ve)**							
ATCC17978[10]	PE whole cell<br>PG whole cell<br>CL whole cell				LC-MS	G56 minimal	PG – 16:0/18:3, 18:1/18:3, 16:0/22:6<br>PE – 16:0/18:3, 18:1/18:3, 16:0/22:6, 18:1/22:6
H01-N[11]	PE CM<br>PE OM<br>L-PE CM<br>L-PE outer<br>CL CM<br>CL outer<br>L-CL CM<br>L-CL outer<br>PG CM<br>PG outer	70<br>51<br>3<br>3<br>7<br>19<br>10<br>15<br>10<br>11			TLC/GC	Non-specific broth	Information relating to alkyl chain composition not provided.
***Pseudomonas aeruginosa* (Gram -ve)**							
PAO1[12]	PC whole cell		32-30 *[a]*	5-17 *[b]*	LC-MS	LB	PC – 35:1, 35:2,

	PE whole cell		32-30 *[a]*	5-17 *[b]*				PE – 16:0/17:1, 16:0/19:1, 16:1/19:1, 17:1/18:1, 17:1/19:1,
	PG whole cell		32-30 *[a]*	5-17 *[b]*				PG – 16:0/17:1, 16:0/19:1, 17:1/18:1, 17:1/19:1
	PC whole cell		6-13 *[a]*	33-13 *[b]*				PC - 32:0, 32:1, 34:2
	PE whole cell		6-13 *[a]*	33-13 *[b]*				PE - 16:0/16:0, 16:0/16:1, 16:1/18:1, 18:1/18:1
	PG whole cell		6-13 *[a]*	33-13 *[b]*				PG - 16:0/16:0, 16:0/18:1, 16:1/18:1
								Lipids that were present, but had different profiles: PC - 34:1, PE - 14:0/18:1, 15:0/18:1, 16:0/16:0/18:1, PG - 16:1/19:1, 18:0/18:1
PAO1 (outer membrane)[13]	PE whole cell<br>PG whole cell<br>PC whole cell			60<br>27<br>13	33 *[c]*<br>63 *[c]*<br>3.9 *[c]*	TLC	LB	Information relating to alkyl chain composition not provided.
B-219[14]	PA whole cell<br>PE whole cell<br>PG whole cell	0.1<br>59<br>41				LC-MS/MS	Universal medium	PA – 34:1 (0.1 %)<br>PE – 32:0 (4 %), 32:1 (18 %), 32:2 (2 %), 33:1 (19 %), 33:2 (3 %), 34:1 (8 %), 34:2 (5 %), 36:2 (4 %)<br>PG – 31:2 (3 %), 32:1 (5 %), 32:2 (4 %), 33:1 (19 %), 34:2 (10 %), 35:2 (6 %), 36:2 (2 %)
PAK (MHB)[15]	PC		2.2 *[d]*				MH	PC – 32:0, 32:1, 34:1, 34:2, 36:1
	PE		17.9 *[d]*					PE – 32:1 34:1, 36:2
	PG		6.7 *[d]*					PG – 22:1, 34:1, 36:2
	CL		1.0 *[d]*					CL – 66.2, 70.3
PAK (SCFM)[15]	PC		0 *[d]*				SCFM	PC – 32:0, 32:1, 34:1, 34:2, 36:1
	PE		13.6 *[d]*					PE – 32:1 34:1, 36:2
	PG		7.4 *[d]*					PG – 22:1, 34:1, 36:2
	CL		0.8 *[d]*					CL – 66.2, 70.3
PAK (SCFM-PC)[15]	PC		22.0 *[d]*				SCFM-PC	PC – 32:0, 32:1, 34:1, 34:2, 36:1
	PE		24.9 *[d]*					PE – 32:1 34:1, 36:2
	PG		15.5 *[d]*					PG – 22:1, 34:1, 36:2
	CL		1.1 *[d]*					CL – 66.2, 70.3
Enterobacter cloacae (E. cloacae) **(Gram -ve)**								
E. cloacae (012)[9]	PE whole cell<br>PG whole cell	65<br>35				FAB-MS	Nutrient broth	PE – 30:0 (3.0 %), 32:2 (6.3 %), 32:1 (20.2 %), 32:0 (0.9 %), 33:1 (6.1 %), 33:0 (1.3 %), 34:2 (9.9 %), 34:1 (12.3 %), 35:2 (1.7 %), 35:1 (2.4 %)<br>PG – 27:3 (5.0 %), 28:3 (3.0 %), 29.4 (2.8 %), 29:3 (1.6 %), 30:2 (7.7 %), 31:2 (8 %), 32:3 (1.0 %), 32:2 (3.7 %), 32:1 (0.9 %), 34:1 (1.1%)
E. cloacae (008)[9]	PE whole cell<br>PG whole cell<br>PS whole cell	50.3<br>42<br>7.7				FAB-MS	Nutrient broth	PE – 30:1 (1.4 %), 30:0 (1.5 %), 31:1 (1.1 %) 32:2 (4.3 %), 32:1 (13.8 %), 32:0 (6.1 %), 33:1 (2.6 %), 34:2 (7.3 %), 34:1 (7.4 %), 34:0 (2.2 %), 35:2 (1.2 %), 35:1 (0.8 %), 38:0 (0.6 %)<br>PG – 27:3 (2.1 %), 28:3 (0.7 %), 29:4 (1.5 %), 29:3 (1.9 %), 30:2 (6.6 %), 30:1 (0.7 %), 31:2 (7.6 %), 32:3 (0.5 %), 32:2 (5.9 %), 32:1 (2.3 %), 33:3 (0.8 %), 33:2 (1.6 %), 33:1 (1.4 %), 34:3 (2.8 %), 34:2 (2.3 %), 34:1 (3.3 %)<br>PS – 34:1 (4.1 %), 32:0 (0.4 %), 33:0 (3.2 %)
E. cloacae S$_{w1}$[16]	PE CM			73		TLC	Soy broth	Information relating to alkyl chain composition not provided.

	PE OM		77			
	L-PE CM		0.4			
	L-PE OM		9			
	PG CM		23			
	PG OM		6			
	CL CM		3.6			
	CL OM		8			
E. cloacae AZT-R[16]	PE CM		80	TLC	Soy broth	Information relating to alkyl chain composition not provided.
	PE OM		7			
	L-PE CM		1			
	L-PE OM		10			
	PG CM		17			
	PG OM		5			
	CL CM		2			
	CL OM		9			
E. cloacae AMA-R[16]	PE CM		79	TLC	Soy broth	Information relating to alkyl chain composition not provided.
	PE OM		82			
	L-PE CM		1			
	L-PE OM		6			
	PG CM		17			
	PG OM		5			
	CL CM		2			
	CL OM		6			
Bacillus subtilis **(Gram +ve)**						
I'1a[17]	PG whole cell	70	75	LC-MS/MS	LB	PE – 28:0 (2 %), 29:0 (4 %), 30:0 (6 %), 32:1 (1 %), 32:0 (2 %), 33:0 (2 %)
	PE whole cell	22	17			PG – 29:0 (5 %), 30:0 (38 %), 31:0 (15 %), 32:0 (15 %)
	CL whole cell	4	7			CL – 61:0 (1 %), 62:0 (1 %), 63:0 (1 %), 64:0 (1 %)
	L-PG whole cell	3	2			L-PG – 32:0 (3 %)
DSM 3257[17]	PG whole cell	65	30	LC-MS/MS	LB	PE – 28:0 (4 %), 29:0 (10 %), 30:0 (9 %), 32:1 (5 %), 32:0 (2 %), 33:0 (4 %)
	PE whole cell	35	70			PG – 29:0 (11 %), 30:0 (24 %), 31:0 (9 %), 32:0 (9 %)
	CL whole cell	4	10			CL – 61:0 (1 %), 62:0 (1 %), 63:0 (1 %), 64:0 (1 %)
	L-PG whole cell	2	2			L-PG – 32:0 (2 %)
Escherichia coli **(Gram -ve)**						
B[18]	PE whole cell	75	decreases	TLC/radioactivity	LB	Information relating to alkyl chain composition not provided.
	PG whole cell	20	9			
	CL whole cell	5	50			
BL21AI Api^{r}[19]	PE (odd)		decreased	RP-HPLC-ESI-iontrap-MS	TSB/M9 minimal	PE – 28:0, 30:1, 30:0, 32:1, 33:1, 34:1, 35:1, 34:2, 35:2, 36:2
	PG (odd)		decreased			PG – 30:0, 32:1, 33:1, 34:1, 35:1, 34:2, 35:2, 36:2, 37:2
	Lipid A					No change in quantity of lipid A between wild type and resistant *E. coli*.

[a] – From 0 minutes to 20 minutes.

[b] – From 3 hours to 24 hours.
[c] – Membrane vesicles.
[d] – Relative area under mass spec peak.

Table S2 – Phospholipid membrane composition in cancer samples.

	Phospholipid	Cancer vs control	Method	Control comparison
**Lung cancer**				
NSCLC Tissue	PC - 32:3, 34:1, 36:2, 36:3[20]	Upregulated	MALDI-MS	Adjacent normal lung tissue – 2 cm apart
	SM - 16:0[20]	Upregulated	MALDI-MS	Adjacent normal lung tissue – 2 cm apart
	PC - 40:4, 40:5, 40:6, 40:7, 42:5, 42:6, 42:7, 42:8, 42:9[21]	Upregulated	ESI-MS	Matched distant lung tissue – > 5 cm apart
	PE - 38:3, 40:3, 40:4, 40:5, 40:6, 42:6, 42:7, 42:8, 42:9, 44:12[21]	Upregulated	ESI-MS	Matched distant lung tissue – > 5 cm apart
	PI - 36:4, 38:2, 38:3, 38:4, 40:3, 40:4, 40:5, 42:2[21]	Upregulated	ESI-MS	Matched distant lung tissue – > 5 cm apart
	PS - 42.9, 44.9[21]	Upregulated	ESI-MS	Matched distant lung tissue – > 5 cm apart
	PI - 32:0, 34:0, 34:1, 36:1, 36:2, 38:6 [21]	Downregulated	ESI-MS	Matched distant lung tissue - > 5 cm apart
	PC - 28:1, 32:0, 32:1, 32:3, 34:0, 34:2, 34:3, 34:4, 36:5, 36:6, 40:8, 44:3, 44.9, 44.10[21]	Downregulated	ESI-MS	Matched distant lung tissue – > 5 cm apart
	PE - 32:0, 34:0, 34.3, 34.4, 36.3, 36.4, 36.5, 38.1, 38.7[21]	Downregulated	ESI-MS	Matched distant lung tissue – > 5 cm apart
	PS - 32:0, 32:1, 34:0, 34:1, 34:2, 34:3 36:1, 36:2, 36:3, 36:4, 38:1, 38:2, 38:3, 38:4, 38:5, 38:6, 38:7, 40:1, 40:2, 40:7, 40:8, 42:2, 42:3, 42:4, 42:5, 42:8[21]	Downregulated	ESI-MS	Matched distant lung tissue – > 5 cm apart
	SM - 34:1, 34:2, 36:1, 36:2, 40:1, 42:1, 42:2[21]	Downregulated	ESI-MS	Matched distant lung tissue – > 5 cm apart
	PC - 32:2, 32:3-1, 32:3-2, 33:1, 33:2, 34:3, 34:4, 35:2, 35:3, 36:1, 36:2, 36:3, 36:4-1, 36:4-2, 36:5, 38:1, 38:2, 38:3, 38:4, 38:5, 38:6[22]	Upregulated	LC-MS	Distal noncancerous tissue
	PE - 32:0, 32:1, 34:0, 34:1, 34:2, 36:3, 36:4, 38:1, 38:4, 38:6[22]	Upregulated	LC-MS	Distal noncancerous tissue
	LPA - 18:1(9Z)/0:0[22]	Upregulated	LC-MS	Distal noncancerous tissue
	LPC - 14:0sn-1, 14:0sn-2, 15:0sn-1, 15:0sn-2, 16:0sn-1, 16:0sn-2, 16:1sn-1, 17:0sn-1, 18:0sn-1, 18:1sn-1, 18:1sn-2, 18:2sn-1, 19:0sn-1, 20:1sn-1, 20:1sn-2, 20:5sn-1, 22:4sn-1, 22:4sn-2, 22:5sn-1, 22:6sn-1[22]	Upregulated	LC-MS	Distal noncancerous tissue
	LPE - 16:0sn-1, 16:0sn-2, 16:1sn-1, 18:0sn-1, 18:0sn-2, 18:1sn-1, 18:1sn-2, 18:2sn-2, 20:0sn-1, 20:1sn-1, 20:2sn-1, 20:2sn-2, 20:3sn-1, 20:4sn-2, 20:5sn-1, 22:4sn-2, 22:6sn-1, 22:6sn-2[22]	Upregulated	LC-MS	Distal noncancerous tissue
	Sphingosine-1-phosphate[22]	Upregulated	LC-MS	Distal noncancerous tissue
Tissue	PC - 34:1, 36:1[23]	Upregulated	MS	Adjacent normal tissue
	PA - 36:2[23]	Upregulated	MS	Adjacent normal tissue
	PC - 38:6[23]	Downregulated	MS	Adjacent normal tissue
	PE - 38:4[23]	Downregulated	MS	Adjacent normal tissue
	PI - 38:4[23]	Downregulated	MS	Adjacent normal tissue
	SM - 22:0, 24:1[23]	Downregulated	MS	Adjacent normal tissue
	PC - 16:0/18:1, 34:1/Na^{+}[24]	Upregulated	MSI	Adjacent normal tissue
Adenocarcinoma Tissue	PC - 16:0/18:1, (16:0/16:1)/(16:0/16:0), (16:0/18:1)/(16:0/18:0), (16:0/18:1)/(16:0/16:0), 36:4[25]	Upregulated	LC-MS	Normal lung tissue
	PC - 16:0/16:0[25]	Downregulated	LC-MS	Normal lung tissue

	SM - 42:2[25]	Downregulated	LC-MS	Normal lung tissue
**Breast cancer**				
Cell lines[*e*]	PC - 32:1, 34:0, 38:5, 38:6[26]	Upregulated	HILIC-HPLC/ESI-MS	MCF10A Cell line
	PE - 32:1, 34:0, 34:1, 34:2, 36:0, 36:4, 38:1, 38.5, 38:6, 40:5, 40:6[26]	Upregulated	HILIC-HPLC/ESI-MS	MCF10A Cell line
	PI - 32:0, 32:1, 34:0, 34:1, 38:5, 40:6[26]	Upregulated	HILIC-HPLC/ESI-MS	MCF10A Cell line
	SM - 36:2, 36:3, 41:1[26]	Upregulated	HILIC-HPLC/ESI-MS	MCF10A Cell line
	PE - 34:5[26]	Downregulated	HILIC-HPLC/ESI-MS	MCF10A Cell line
	PI - 38:3[26]	Downregulated	HILIC-HPLC/ESI-MS	MCF10A Cell line
	SM - 40:1[26]	Downregulated	HILIC-HPLC/ESI-MS	MCF10A Cell line
T-47D	PC - 14:0/18:1[26]	Upregulated	TLC, ESI-MS	MCF10A cell line
	PE - 16:1/18:0, 16:0/18:1, 18:0/18:2, 18:1/18/1[26]	Upregulated	TLC, ESI-MS	MCF10A cell line
	PI - 18:1/18:1, 18:2/18:0, 18:0/18:1[26]	Upregulated	TLC, ESI-MS	MCF10A cell line
	CL - 16:1/18:1/18:1/18:2[26]	Upregulated	TLC, ESI-MS	MCF10A cell line
	PC - 18:1/18:1, 18:0/18:1[26]	Downregulated	TLC, ESI-MS	MCF10A cell line
	PI - 22.5/18:0[26]	Downregulated	TLC, ESI-MS	MCF10A cell line
	CL - 16:1/18:1/18:2/20:3, 18:2/18:2/18:2/20:2[26]	Downregulated	TLC, ESI-MS	MCF10A cell line
	SM - 18:1/22:0, 18:1/24.1, 18:1/24:0[26]	Downregulated	TLC, ESI-MS	MCF10A cell line
MDA-MB-231	PC - 14:0/18:1, 16:0/16:1, 18:1/18:1,18:0/18:1[26]	Upregulated	TLC, ESI-MS	MCF10A cell line
	PI - 18:1/18:1, 18:2/18:0, 18:0/18:1, 22.5/18:0[26]	Upregulated	TLC, ESI-MS	MCF10A cell line
	SM - 18:1/22:0, 18:/24:0[26]	Upregulated	TLC, ESI-MS	MCF10A cell line
	PC - 16:1/18:1, 18:1/18:2, 18:1/18:1, 18:2/20:3, 18:0/20:4, 18:0/20:0, 18:1/20:0, 18:1/22:5[27]	Upregulated	DI-MS	MCF10A cell line
	PE - 17:0/19:0, 18:1/20:4, 18:1/22:4[27]	Upregulated	DI-MS	MCF10A cell line
	PI - 16:0/16:1, 16:1/18:1, 16:0/18:1, 16:0/22:6, 18:1/20:4, 18:0/20:4, 18:0/20:3[27]	Upregulated	DI-MS	MCF10A cell line
	PS - 16:1/18:1, 16:0/18:0, 16:0/18:1, 17:0/18:1, 18:0/18:0, 18:1/20:0, 18:0/22:6, 18:1/22:4, 18:1/22:0[27]	Upregulated	DI-MS	MCF10A cell line
	PE - 18:1/18:1[27]	Downregulated	DI-MS	MCF10A cell line
	PG - 16:0/18:1, 18:1/18:1[27]	Downregulated	DI-MS	MCF10A cell line
	PI - 18:1/18:1, 18:0/18:1[27]	Downregulated	DI-MS	MCF10A cell line
MCF-7	PC - 16:1/18:1, 18:1/18:2, 18:1/18:1, 18:2/20:3, 18:0/20:4, 18:1/20:1, 18:1/20:0, 18:1/22:5[27]	Upregulated	DI-MS	MCF10A cell line
	PE - 18:1/18:1, 17:0/19:0, 18:1/20:4, 18:1/22:4[27]	Upregulated	DI-MS	MCF10A cell line
	PG - 16:0/18:1, 18:1/18:1[27]	Upregulated	DI-MS	MCF10A cell line
	PI - 16:0/16:1, 16:1/18:1, 16:0/18:1, 18:1/20:4[27]	Upregulated	DI-MS	MCF10A cell line
	PS - 16:1/18:1, 16:0/18:0, 16:0/18:1, 17:0/18:1, 18:0/18:0, 18:1/20:0, 18:0/22:6, 18:1/22:4, 18:1/22:0[27]	Upregulated	DI-MS	MCF10A cell line
	PI - 18:1/18:1, 18:0/20:4, 18:0/20:3[27]	Downregulated	DI-MS	MCF10A cell line
	PS - 18:1/18:2, 18:0/18:1, 18:1/22:1[27]	Downregulated	DI-MS	MCF10A cell line

Tissue	PC[28]	Upregulated	UPLC-MS	Adjacent normal tissue
	PE[28]	Upregulated	UPLC-MS	Adjacent normal tissue
	PI[28]	Upregulated	UPLC-MS	Adjacent normal tissue
	SM[28]	Upregulated	UPLC-MS	Adjacent normal tissue
Tissue	PC - 38:6[29]	Upregulated	HILIC-HPLC/ESI-MS	Normal breast tissue
	PE - 34:1, PE 36:1[29]	Upregulated	HILIC-HPLC/ESI-MS	Normal breast tissue
	PI - 36:4[29]	Upregulated	HILIC-HPLC/ESI-MS	Normal breast tissue
	SM - 34:1[29]	Downregulated	HILIC-HPLC/ESI-MS-MALDI- MS	Normal breast tissue
	PC - 32:0, 34:1[30]	Upregulated	MALDI-MS	Adjacent normal breast tissue
	SM - d18:1/16:0[30]	Upregulated	MALDI-MS	Adjacent normal breast tissue
	Sphingosine, sphingosine-1-phosphate, dihydro-sphingosine, dihydro-sphingosine-1-phosphate[31]	Upregulated	LC-ESI-MS/MS	Normal breast tissue
	PC - 32:1, 34:1, 36:1[23]	Upregulated	MS	Adjacent normal tissue
	PA - 36:2[23]	Upregulated	MS	Adjacent normal tissue
	PC - 38:4, 38:6[23]	Downregulated	MS	Adjacent normal tissue
	PA - 38:3, 40:5[23]	Downregulated	MS	Adjacent normal tissue
	PE - 38:4[23]	Downregulated	MS	Adjacent normal tissue
	PI - 38:4[23]	Downregulated	MS	Adjacent normal tissue
	SM - 22:0, 24:1[23]	Downregulated	MS	Adjacent normal tissue
**Prostate cancer**				
Cell lines DU145, 22RV1, LNCaP	PC - 32:1, 34:1[32]	Upregulated *[f]*	LC-ESI-MS/MS	Cell line PNT1a
	PC - 36:2[32]	Upregulated *[g]*	LC-ESI-MS/MS	Cell line PNT1a
	PE - 18:1/16:0[32]	Upregulated *[h]*	LC-ESI-MS/MS	Cell line PNT1a
	PE - 18:1/18:1[32]	Upregulated *[i]*	LC-ESI-MS/MS	Cell line PNT1a
	SM - 18:1/20:0[32]	Upregulated *[f]*	LC-ESI-MS/MS	Cell line PNT1a
	SM - 18:1/16:0[32]	Upregulated *[h]*	LC-ESI-MS/MS	Cell line PNT1a
	SM - 18:1/22:0[32]	Upregulated *[g]*	LC-ESI-MS/MS	Cell line PNT1a
	PC - 32:1, 34:1[32]	Downregulated *[j]*	LC-ESI-MS/MS	Cell line PNT1a
	PC - 36:2[32]	Downregulated *[k]*	LC-ESI-MS/MS	Cell line PNT1a
	PE - 18:1/16:0[32]	Downregulated *[l]*	LC-ESI-MS/MS	Cell line PNT1a
	SM - 18:1/20:0, 18:1/22:0[32]	Downregulated *[j]*	LC-ESI-MS/MS	Cell line PNT1a
Tissue	PI - 18:0/18:1, 18:0/20:3, 18:0/20:2[33]	Upregulated	High res MALDI-MSI	Adjacent benign epithelium
	LPC - 16:0/OH[34]	Downregulated	High res MALDI-MSI	Adjacent benign epithelium
	PE - 44:7, 34:2[35]	Upregulated	DESI-MSI	Benign prostate tissue
	PI - 37:2[35]	Upregulated	DESI-MSI	Benign prostate tissue
	PI - 37:6, 33:1[35]	Downregulated	DESI-MSI	Benign prostate tissue
	LPE - 16:0, 18:0, 18:1[35]	Downregulated	DESI-MSI	Benign prostate tissue

	PC - 16:0/14:0, 16:0/16:1, 16:0/18:2, 16:0/18:1, 16:0/18:0, 18:1/18:2, 18:1/18:1, 18:0/18:1, 18:1/20:4, 18:1/20:218:1/20:1, 18:1/20:0[35]	Upregulated	ESI-QqQ/MS	Tissue from healthy males
	SM - d18:1/24:2, d18:1/24:0[35]	Upregulated	ESI-QqQ/MS	Tissue from healthy males
	PC - 18:0/22:5[35]	Downregulated	MALDI-ToF/MS	Tissue from healthy males
**Colorectal cancer**				
Tissue	PA - 31:0[36]	Upregulated	REIMS	Normal adjacent mucosa
	PG - 36:1[36]	Upregulated	REIMS	Normal adjacent mucosa
	PS - 41:0, 44:6, 44:8[36]	Upregulated	REIMS	Normal adjacent mucosa
	PS - 43:5[36]	Downregulated	REIMS	Normal adjacent mucosa
	PA - 36:2[23]	Upregulated	MS	Adjacent normal tissue
	PC - 32:1, PC 34:1[23]	Upregulated	MS	Adjacent normal tissue
	PA - 38:3, PA 40:5[23]	Downregulated	MS	Adjacent normal tissue
	PC - 38:4[23]	Downregulated	MS	Adjacent normal tissue
	PE - 38:4[23]	Downregulated	MS	Adjacent normal tissue
	PI - 38:4[23]	Downregulated	MS	Adjacent normal tissue
	SM - 22:0, 24:1[23]	Downregulated	MS	Adjacent normal tissue
	PC[37]	Upregulated	HPLC	Adjacent normal tissue
	PC - 16:0/18:1[38]	Upregulated	MALDI-MSI	Adjacent colorectal mucosa and tumour remote tissue – 10 cm apart
	LPC - 16:0, 18:1[38]	Upregulated	MALDI-MSI	Adjacent colorectal mucosa and tumour remote tissue – 10 cm apart
	PC - 16:0/16:1[39]	Upregulated	Imaging MS	Adjacent non-neoplastic mucosa
	PC[40]	Upregulated	MS	Adjacent normal mucosa
	PE[40]	Upregulated	MS	Adjacent normal mucosa
	PC, PE , PI[41]	Upregulated	MRS	Adjacent normal mucosa – 4 cm or greater apart
	PS[41]	Downregulated	MRS	Adjacent normal mucosa – 4 cm or greater apart
	PC - 26:0, 28:1, 30:0, 31:2, 31:4, 32:1, 34:1[42]	Upregulated	LC-MS	Tumour adjacent tissue
	PE - 28:1, 31:4, 33:4[42]	Upregulated	LC-MS	Tumour adjacent tissue
	PG - 37:6[42]	Upregulated	LC-MS	Tumour adjacent tissue
	PS - 16:0, 28:2[42]	Upregulated	LC-MS	Tumour adjacent tissue
	LPC - 24:0[42]<br>LPE - 16:1, 18:2[42]	Upregulated	LC-MS	Tumour adjacent tissue
	LPG - 20:4, 21:0[42]	Upregulated	LC-MS	Tumour adjacent tissue
	SM - 24:2, 39:2[42]	Upregulated	LC-MS	Tumour adjacent tissue
	Sphingosine - 14:2, 18:3[42]	Upregulated	LC-MS	Tumour adjacent tissue
Cells isolated from tumour tissue	PC - 32:0, 32:1, 36:5, 38:4[43]	Upregulated	LC-MS/MS	Cells isolated from tumour adjacent normal tissue
	LPC - 14:0, 22:0, 24:0, 26:0, 16:1, 22:1, 24:1, 20:3, 20:4, 22:4, 22:5, 22:6[43]	Upregulated	LC-MS/MS	Cells isolated from tumour adjacent normal tissue

	LPE - 18:0, 22:0, 24:0, 16:1, 22:1, 24:1, 20:3, 20:4, 22:4, 22:5, 22:6[43]	Upregulated	LC-MS/MS	Cells isolated from tumour adjacent normal tissue
	LPS - 16:1, 20:3[43]	Upregulated	LC-MS/MS	Cells isolated from tumour adjacent normal tissue
**Colorectal Liver Metastasis**				
Tumour tissue	PC - 36:2-16:0/20:2, 38:3-18:1/20:2, 38:2-18:1/20:1, 36:2-16:1/20:1[44]	Upregulated	LC-ESI-MS	Normal liver parenchyma
	PE - 40:1-18:1/22:0, 38:3-18:1/20:2, 38:4-18:2/20:2, 34:1-16:1/18:0[44]	Upregulated	LC-ESI-MS	Normal liver parenchyma
	PS - 36:2-18:1/18:1[44]	Upregulated	LC-ESI-MS	Normal liver parenchyma
	SM - 34:1-d18:1/16:0[44]	Upregulated	LC-ESI-MS	Normal liver parenchyma
	PC - 38:6-16:0/22:6[44]	Downregulated	LC-ESI-MS	Normal liver parenchyma
	PE - 38:7-16:1/22:6, 34:3-16:0/18:3, 40:7-18:1/22:6, 38:6-16:0/22:6, 36:5-16:0/20:5, 36:4-16:0/20:4, 40:6-18:0/22:6[44]	Downregulated	LC-ESI-MS	Normal liver parenchyma
	PI - 36:1-16:0/20:1[44]	Downregulated	LC-ESI-MS	Normal liver parenchyma
	PS - 40:7-18:1/22:6[44]	Downregulated	LC-ESI-MS	Normal liver parenchyma
**Ovarian cancer**				
Tissue sample	PC - 32:3 , 34:1, 36:2[45]	Upregulated	MALDI-MS	Adjacent normal tissue
	PI - 38:4[45]	Upregulated	MALDI-MS	Adjacent normal tissue
**Urothelial cancer of the bladder**				
Tumour tissue	PC[46]	Upregulated	LC-MS	Benign adjacent tissue, normal bladder tissue
	PE[46]	Upregulated	LC-MS	Benign adjacent tissue, normal bladder tissue
	PI[46]	Upregulated	LC-MS	Benign adjacent tissue, normal bladder tissue
	PS[46]	Upregulated	LC-MS	Benign adjacent tissue, normal bladder tissue
**Pancreatic cancer**				
Ductal adenocarcinoma tissue	PS[47] (detected on the cell surface)	Upregulated	Annexin V-FITC Staining, Flow Cytometry	Adjacent normal tissue
**Gastric cancer**				
Tissue	PC - 16:0/18:0[48]	Upregulated	MALDI-MSI	Adjacent non-neoplastic mucosa
	LPC - 16:0[48]	Downregulated	MALDI-MSI	Adjacent non-neoplastic mucosa
	PC - 32:1, 34:1, 36:1[23]	Upregulated	MS	Adjacent normal tissue
	PA - 36:2[23]	Upregulated	MS	Adjacent normal tissue
	PC - 38:4, 38:6[23]	Downregulated	MS	Adjacent normal tissue
	PA - 38:3, 40:5[23]	Downregulated	MS	Adjacent normal tissue
	PE - 38:4[23]	Downregulated	MS	Adjacent normal tissue
	PI - 38:4[23]	Downregulated	MS	Adjacent normal tissue
	SM - 22:0, 24:1[23]	Downregulated	MS	Adjacent normal tissue
**Glioblastoma**				

Cell line A172 (Single cell analysis)	PC - 32:2/32:1, 32:2/32:0, 32:1/32:0, 33:2/33:1, 33:2/33:0, 33:1/33:0, 34:2/34:1, 34:2/34:0, 34:1/34:0[49]	Upregulated	Pico-ESI-MS	Normal human astrocyte cell line HA
	PE - 20:1[49]	Upregulated	Pico-ESI-MS	(Single cell analysis)
**Oesophageal Cancer**				
Adenocarcinoma tissue	PE - 38:1, 38:5[50]	Upregulated	DESI-MSI	Matched healthy oesophageal epithelium - <5 cm apart
	PG - 40:6, 38:6, PG 38:5, 40:8, 40:7, 36:4, 34:2, 36:3, 36:0, 36:2[50]	Upregulated	DESI-MSI	Matched healthy oesophageal epithelium - <5 cm apart
	PI - 37:4, 40:5, 40:4, 38:5[50]	Upregulated	DESI-MSI	Matched healthy oesophageal epithelium - <5 cm apart
	PS - 44:9, 42:8[50]	Upregulated	DESI-MSI	Matched healthy oesophageal epithelium - <5 cm apart
	PA - 38:1[50]	Upregulated	DESI-MSI	Matched healthy oesophageal epithelium - <5 cm apart
	PE - 34:1[50]	Downregulated	DESI-MSI	Matched healthy oesophageal epithelium - <5 cm apart
	PS - 36:2[50]	Downregulated	DESI-MSI	Matched healthy oesophageal epithelium - <5 cm apart
	PA - 36:2[50]	Downregulated	DESI-MSI	Matched healthy oesophageal epithelium - <5 cm apart
Tissue	PC - 32:1, 34:1, 36:1[23]	Upregulated	MS	Adjacent normal tissue
	PA - 36:2[23]	Upregulated	MS	Adjacent normal tissue
	PC - 38:4, 38:6[23]	Downregulated	MS	Adjacent normal tissue
	PA - 38:3, 40:5[23]	Downregulated	MS	Adjacent normal tissue
	PE - 38:4[23]	Downregulated	MS	Adjacent normal tissue
	PI - 38:4[23]	Downregulated	MS	Adjacent normal tissue
	SM - 22:0, 24:1[23]	Downregulated	MS	Adjacent normal tissue
**Thyroid cancer**				
Tissue	PC - 16:0/18:1, 16:0/18:2[51]	Upregulated	MSI	Adjacent normal tissue
	SM - d18:0/16:1[51]	Upregulated	MSI	Adjacent normal tissue
	PC - 34:1, 38:6[23]	Upregulated	MS	Adjacent normal tissue
	PA - 36:2[23]	Upregulated	MS	Adjacent normal tissue
	PA - 38:3, 40:5[23]	Downregulated	MS	Adjacent normal tissue
	PE - 38:4[23]	Downregulated	MS	Adjacent normal tissue
	PI 38:4[23]	Downregulated	MS	Adjacent normal tissue
	SM - 22:0, 24:1[23]	Downregulated	MS	Adjacent normal tissue
**Hepatocellular carcinoma**				
Tissue	PC - 30:0[52]	Upregulated	ESI-MS	Benign hepatic tissue
	PC - 34:2[52]	Downregulated	ESI-MS	Benign hepatic tissue
	PE - 34:2[52]	Downregulated	ESI-MS	Benign hepatic tissue
	SM - 24:0[52]	Downregulated	ESI-MS	Benign hepatic tissue
	PC - 34:0, 32:0, 42:2, 34:3, 32:2, 44:3[53]	Upregulated	UPLC-MS	Normal liver tissue
	PC - 26:0, 34:2, 38:6, 40:5[53]	Downregulated	UPLC-MS	Normal liver tissue
**Renal cell carcinoma**				

Tissue	PC - 14:0/16:0, 14:0/18:2, 16:0/16:0, 16:0/16:1, 16:1/16:1, 16:0/18:0, 16:1/18:2, 17:0/18:2, 18:0/18:2, 18:2/18:2, 18:0/20:3, 18:0/20:4, 20:1/18:2, 20:2/18:1[54]	Upregulated	LC-TOF/MS	Surrounding normal renal cortex
	PC - 16:0/18:1, 16:0/18:2, 16:0/20:4, 16:0/20:5, 18:1/20:4, 16:0/22:5, 16:0/22:6[54]	Downregulated	LC-TOF/MS	Surrounding normal renal cortex

[*e*] – Nine breast cancer cell lines (ZR-75-1, T-47D, MCF7, MDA-MB-231, MDA-MB-453, MDA-MB-468, SK-BR-3, BT-474 and BT-549).
[*f*]- Upregulated in LNCaP, 22RV1
[*g*]- Upregulated in 22RV1
[*h*]- Upregulated in LNCaP
[*i*]- Upregulated in all 3 cell lines
[*j*]- Downregulated in DU145
[*k*]- Downregulated in LNCaP, DU145
[*l*]- Downregulated in 22RV1

Abbreviations

2D TLC – Two-dimensional thin layer chromatography.

AB – Agrobacterium medium, derivative of M9 minimal medium.

BHI – Brain heart infusion.

DESI – Desorption electron spray ionization.

DESI-MS - Desorption electron spray ionization mass spectrometry.

DESI-IMS - Desorption electron spray ionization imaging mass spectrometry.

DI-ESI(±)-FTICR MS – Direct-infusion positive and negative ion electrospray ionization Fourier transform ion cyclotron resonance mass spectrometry.

DI MS – Direct-infusion electrospray ionization mass spectrometry.

ESI-MS – Electron spray ionization mass spectrometry.

ESI-MS/MS –Electron spray ionization tandem mass spectrometry.

FAB-MS – Fast atom bombardment mass spectrometry.

GC – Gas chromatography.

GC-MS – Gas chromatography coupled with mass spectrometry.

High res – High resolution.

HILIC-HPLC/ESI-MS – Hydrophilic interaction chromatography coupled with high performance liquid chromatography coupled with electron spray ionization mass spectrometry.

HILIC-HPLC/ESI-MS-MALDI-MS – Hydrophilic interaction chromatography coupled with high performance liquid chromatography coupled with electron spray ionization tandem mass spectrometry and matrix-assisted laser desorption.

HPLC – High performance liquid chromatography.

IHC – Immunohistochemistry.

MSI – Mass spectrometry imaging.

LC/ESI-MS/MS – Liquid chromatography coupled with electron spray ionization tandem mass spectrometry.

LC-MS – Liquid chromatography coupled with mass spectrometry.

LC-MS/MS - Liquid chromatography coupled with tandem mass spectrometry.

LC-TOF/MS – Liquid chromatography coupled with time-of-flight mass spectrometry.

MALDI-FTICR-MS – Matrix-assisted laser desorption/ionization Fourier transform ion cyclotron mass spectrometry.

MALDI-MS – Matrix-assisted laser desorption/ionization mass spectrometry.

MALDI-MSI – Matrix-assisted laser desorption mass spectrometry imaging.

MHA – Mueller-Hinton agar.

MS – Mass spectrometry.

MRS – Magnetic resonance spectroscopy.

nLC/ESI-MS/MS – Nano-scale liquid chromatography coupled with electron spray ionization tandem mass spectrometry.

NMR – Nuclear magnetic resonance spectroscopy.

Non-specific broth (per L) – $(NH_4)_2SO_4$ 2 g; KH_2PO_4 4 g; Na_2HPO_4 $7H_20$ 4 g; $MgSO_4$ $7H_2O$ 0.2 g; $CaCl_2$ $2H_2O$, 0.001 g; and $FeSO_4$ $7H_2O$, 0.001 g, pH 7.8.

REIMS – Rapid evaporative ionization mass spectrometry.

RP-HILIC – Reverse phase hydrophilic interaction liquid chromatography.

RP-HPLC-ESI-iontrap-MS – Reverse phase high performance liquid chromatography coupled with electron spray ionization, iontrap mass spectrometry.

RRLC-MS – Rapid resolution liquid chromatography coupled with mass spectrometry.

Synthetic broth 1 (per L) – Na_2HPO_4 $12H_2O$ 13.9 g; KH_2PO_4 2.1 g; NH_4Cl 1.0 g; $CaCl_2$ 0.0146 g; $MgSO_4$ 0.22 g; $FeCl_3.4H_2O$ 0.24 mg; sodium lactate 2.5 g; peptone 5 g; yeast extract 1.0 g and NaCl 0.5 g, 50 g or I00 g.

Synthetic media 2 (per L) – 19 mM KCl, 0.49 mM K_2HPO_4, 79 mM NaCl, 20 mM NH_4Cl, 1.4 mM Na_2SO_4, 0.1 mM adenine, 0.1 mM xanthine, 0.1 mM uracil, 1.8 mM alanine, 1.4 mM arginine, 0.75 mM asparagine, 1.5 mM cysteine, 0.75 mM glutamic acid, 0.3 mM glycine, 1.1 mM histidine, 2 mM isoleucine, 4 mM leucine, 3.5 mM lysine, 1.1 mM methionine, 0.09 mM phenylalanine, 4.6 mM proline, 2.8 mM serine, 1.9 mM threonine, 0.08 mM tyrosine, 2.5 mM valine, and 0.1 M tris(hydroxymethyl) aminomethane, pH 7.4.

Synthetic media 3 (per L) – Substrate Substrate (glucose or succinate) 5 g; NH_4NO_3 6 g; K_2HPO_4 5 g; KH_2PO_4 2 g; 0.5 mL of 1 % $CaCl_2$ $2H_2O$ solution in water; 1 mL of trace elements solution containing (g/L): $MnCl_2$ $.4H_2O$ 0.004 g; $CuSO_4$ $5H_2O$ 2.0 g; $ZnSO_4$ $7H_2O$ 2.2 g; ammonium molybdate, 0.22 g; and $FeSO_4$ $7H_2O$ 1.0 mL of 1.0 %, pH 6).

TLC – Thin layer chromatography.

TSB – Tryptic soy broth (enriched with chloramphenicol or erythromycin).

UHPLC-ESI-TOF/MS – Ultra high-performance liquid chromatography coupled with electron spray ionization time-of-flight mass spectrometry.

UPLC-ESI-MS - Ultra high-performance liquid chromatography coupled with electron spray ionization mass spectrometry.

UHPLC-MS – Ultra high-performance liquid chromatography coupled with mass spectrometry.

UPLC – Ultra high-performance liquid chromatography.

References

1 N. N. Mishra, A. S. Bayer, T. T. Tran, Y. Shamoo, E. Mileykovskaya, W. Dowhan, Z. Guan and C. A. Arias, *PLoS One*, 2012, **7**, e43958–e43958.

2 H. U. KOCH, R. HAAS and W. FISCHER, *Eur. J. Biochem.*, 1984, **138**, 357–363.

3 Z. DeMars, V. K. Singh and J. L. Bose, *J. Bacteriol., 2020, **202**(14):e00128-20.*

4 N. N. Mishra, S.-J. Yang, A. Sawa, A. Rubio, C. C. Nast, M. R. Yeaman and A. S. Bayer, *Antimicrob. Agents Chemother.*, 2009, **53**, 2312–2318.

5 A. Okabe, Y. Hirai, H. Hayashi and Y. Kanemasa, *Biochim. Biophys. Acta (BBA)-Lipids Lipid Metab.*, 1980, **617**, 28–35.

6 K. Yasuhiro, Y. Tieko and H. Hideo, *Biochim. Biophys. Acta Lipids Lipid Metab.*, 1972, **280**, 444–450.

7 P. H. Ray and D. C. White, *J. Bacteriol.*, 1972, **109**, 668–677.

8 M. K. Wassef, *Lipids*, 1976, **11**, 364–369.

9 H. S. Aluyi, V. Boote, D. B. Drucker, J. M. Wilson and Y. H. Ling, *J. Appl. Bacteriol.*, 1992, **73**, 426–432.

10 A. E. Eder, S. A. Munir, C. R. Hobby, D. M. Anderson, J. L. Herndon, A. W. Siv, S. J. K. Symes and D. K. Giles, *Microbiology*, 2017, **163**, 1626–1636.

11 C. C. Scott, S. R. Makula and W. R. Finnerty, *J. Bacteriol.*, 1976, **127**, 469–480.

12 C. Le Sénéchal, M. Crouzet, P. Costaglioli, C. Barthe, C. Buré and S. Vilain, *Lipids*, 2019, **54**, 519–529.

13 T. Yosuke, I. Aya, S. Motoyuki, I. Sosaku, T. Naoki, N.-K. Toshiaki, U. Hiroo and N. Nobuhiko, *Biotechnol. Biochem.*, 2011, **75**, 605–607.

14 P. Bernat, P. Siewiera, A. Soboń and J. Długoński, *World J. Microbiol. Biotechnol.*, 2014, **30**, 2343–2350.

15 E. Deschamps, A. Schaumann, I. Schmitz-Afonso, C. Afonso, E. Dé, C. Loutelier-Bourhis and S. Alexandre, *Biochim. Biophys. Acta Biomembr.*, 2021, **1863**, 183482.

16 R. Aggeler, R. L. Then and R. Ghosh, *J. Gen. Microbiol.*, 1987, **133**, 3383–3392.

17 P. Bernat, K. Paraszkiewicz, P. Siewiera, M. Moryl, G. Płaza and J. Chojniak, *World J. Microbiol. Biotechnol.*, 2016, **32**, 1–13.

18 S. Hiraoka, H. Matsuzaki and I. Shibuya, *FEBS Lett.*, 1993, **336**, 221–224.

19 R. Schmidt, D. Yonghong and R. Hoffmann, *Diagn. Microbiol. Infect. Dis.*, 2018, **90**, 316–323.

20 G. K. Lee, H. S. Lee, Y. S. Park, J. H. Lee, S. C. Lee, J. H. Lee, S. J. Lee, S. R. Shanta, H. M. Park, H. R. Kim, I. H. Kim, Y. H. Kim, J. I. Zo, K. P. Kim and H. K. Kim, *Lung Cancer*, 2012, **76**, 197–203.

21 E. Marien, M. Meister, T. Muley, S. Fieuws, S. Bordel, R. Derua, J. Spraggins, R. de Plas, J. Dehairs, J. Wouters, M. Bagadi, H. Dienemann, M. Thomas, P. A. Schnabel, R. M. Caprioli, E. Waelkens and J. V Swinnen, *Int. J. Cancer*, 2015, **137**, 1539–1548.

22 L. You, Y. Fan, X. Liu, S. Shao, L. Guo, H. A. A. Noreldeen, Z. Li, Y. Ouyang, E. Li, X. Pan, T. Liu, X. Tian, F. Ye, X. Li and G. Xu, *J. Proteome Res.*, 2020, **19**, 3750–3760.

23 S. Guo, Y. Wang, D. Zhou and Z. Li, *Sci. Rep.*, 2014, **4**, 5959.

24 M. Zhang, J. He, T. Li, H. Hu, X. Li, H. Xing, J. Wang, F. Yang, Q. Ma, B. Liu, C. Tang, Z. Abliz and X. Liu, *Front. Oncol.*, 2019, **0**, 804.

25 Y. Muranishi, T. Sato, S. Ito, J. Satoh, A. Yoshizawa, S. Tamari, Y. Ueda, Y. Yutaka, T. Menju, T. Nakamura and H. Date, *Sci. Rep.*, 2019, **9**, 8916.

26 M. L. Dória, C. Z. Cotrim, C. Simões, B. Macedo, P. Domingues, M. R. Domingues and L. A. Helguero, *J Cell Physiol*, 2013, **228**, 457–468.

27 H. Y. Kim, K. M. Lee, S. H. Kim, Y. J. Kwon, Y. J. Chun and H. K. Choi, *Oncotarget*, 2016, **7**, 67111–67128.

28 M. Hilvo, C. Denkert, L. Lehtinen, B. Müller, S. Brockmöller, T. Seppänen-Laakso, J. Budczies, E. Bucher, L. Yetukuri, S. Castillo, E. Berg, H. Nygren, M. Sysi-Aho, J. L. Griffin, O. Fiehn, S. Loibl, C. Richter-Ehrenstein, C. Radke, T. Hyötyläinen, O. Kallioniemi, K. Iljin and M. Orešič, *Cancer Res.*, 2011, **71**, 3236–3245.

29 E. Cífková, M. Holčapek, M. Lísa, D. Vrána, J. Gatěk and B. Melichar, *Anal. Bioanal. Chem.*, 2015, **407**, 991–1002.

30 S. Kang, A. Lee, Y. S. Park, S. C. Lee, S. Y. Park, S. Y. Han, K. P. Kim, Y. H. Kim, C. W. Yoo and H. K. Kim, *Int. J. Gynecol. Cancer*, 2011, **21**, 1566–1572.

31 J. Tsuchida, M. Nagahashi, M. Nakajima, E. Katsuta, O. M. Rashid, Q. Qi, L. Yan, S. Okuda, K. Takabe and T. Wakai, *J. Surg. Res.*, 2020, **256**, 645–656.

32 A. Sorvina, C. A. Bader, C. Caporale, E. A. Carter, I. R. D. Johnson, E. J. Parkinson-Lawrence, P. V. Simpson, P. J. Wright, S. Stagni, P. A. Lay, M. Massi, D. A. Brooks and S. E. Plush, *Oncotarget*, 2018, **9**, 35541.

33 T. Goto, N. Terada, T. Inoue, K. Nakayama, Y. Okada, T. Yoshikawa, Y. Miyazaki, M. Uegaki, S. Sumiyoshi, T. Kobayashi, T. Kamba, K. Yoshimura and O. Ogawa, *PLoS One*, 2014, **9**, e90242.

34 T. Goto, N. Terada, T. Inoue, T. Kobayashi, K. Nakayama, Y. Okada, T. Yoshikawa, Y. Miyazaki, M. Uegaki and N. Utsunomiya, *Prostate*, 2015, **75**, 1821–1830.

35 N. Morse, T. Jamaspishvili, D. Simon, P. G. Patel, K. Y. M. Ren, J. Wang, R. Oleschuk, M. Kaufmann, R. J. Gooding and D. M. Berman, *Lab. Investig.*, 2019, **99**, 1561–1571.

36 J. Alexander, L. Gildea, J. Balog, A. Speller, J. McKenzie, L. Muirhead, A. Scott, C. Kontovounisios, S. Rasheed, J. Teare, J. Hoare, K. Veselkov, R. Goldin, P. Tekkis, A. Darzi, J. Nicholson, J. Kinross and Z. Takats, *Surg. Endosc.*, 2017, **31**, 1361–1370.

37 I. Dobrzyńska, B. Szachowicz-Petelska, S. Sulkowski and Z. Figaszewski, *Mol. Cell. Biochem.*, 2005, **276**, 113–119.

38 R. Mirnezami, K. Spagou, P. A. Vorkas, M. R. Lewis, J. Kinross, E. Want, H. Shion, R. D. Goldin, A. Darzi, Z. Takats, E. Holmes, O. Cloarec and J. K. Nicholson, *Mol. Oncol.*, 2014, **8**, 39–49.

39 N. Kurabe, T. Hayasaka, M. Ogawa, N. Masaki, Y. Ide, M. Waki, T. Nakamura, K. Kurachi, T. Kahyo, K. Shinmura, Y. Midorikawa, Y. Sugiyama, M. Setou and H. Sugimura, *Cancer Sci.*, 2013, **104**, 1295–1302.

40 D. A. Dueck, M. Chan, K. Tran, J. T. Wong, F. T. Jay, C. Littman, R. Stimpson and P. C. Choy, *Mol. Cell Biochem.*, 1996, **162**, 97–103.

41 T. E. Merchant, J. N. Kasimos, P. W. de Graaf, B. D. Minsky, L. W. Gierke and T. Glonek, *Int. J. Color. Dis.*, 1991, **6**, 121–126.

42 A. Pakiet, K. Sikora, J. Kobiela, O. Rostkowska, A. Mika and T. Sledzinski, *Lipids Health Dis.*, 2021, **20**, 85.

43 J. Hofmanová, J. Slavík, M. Ciganek, P. Ovesná, Z. Tylichová, M. Karasová, O. Zapletal, N. Straková, J. Procházková, J. Bouchal, Z. Kolář, J. Ehrmann, M. Levková, Z. Hušková, P. Skalický, A. Kozubík, M. Machala and J. Vondráček, *Int. J. Mol. Sci.*, 2021, **22(**13), 6650.

44 S. Kiritani, K. Yoshimura, J. Arita, T. Kokudo, H. Hakoda, M. Tanimoto, T. Ishizawa, N. Akamatsu, J. Kaneko, S. Takeda and K. Hasegawa, *BMC Cancer 2021 211*, 2021, **21**, 1–9.

45 S. Kang, A. Lee, Y. S. Park, S. C. Lee, S. Y. Park, S. Y. Han, K. P. Kim, Y. H. Kim, C. W. Yoo and H. K. Kim, *Int. J. Gynecol. Cancer*, 2011, **21**, 1566–1572.

46 D. W. B. Piyarathna, T. M. Rajendiran, V. Putluri, V. Vantaku, T. Soni, F. C. von Rundstedt, S. R. Donepudi, F. Jin, S. Maity, C. R. Ambati, J. Dong, D. Gödde, S. Roth, S. Störkel, S. Degener, G. Michailidis, S. P. Lerner, S. Pennathur, Y. Lotan, C. Coarfa, A. Sreekumar and N. Putluri, *Eur. Urol. Focus*, 2018, **4**, 907–915.

47 H. W. Davis, A. Kaynak, S. D. Vallabhapurapu and X. Qi, *World J. Gastrointest. Oncol.*, 2021, **13**, 550.

48 T. Uehara, H. Kikuchi, S. Miyazaki, I. Iino, T. Setoguchi, Y. Hiramatsu, M. Ohta, K. Kamiya, Y. Morita, H. Tanaka, S. Baba, T. Hayasaka, M. Setou and H. Konno, *Ann Surg Oncol*, 2016, **23** Suppl 2, S206-13.

49 X.-C. Zhang, Q. Zang, H. Zhao, X. Ma, X. Pan, J. Feng, S. Zhang, R. Zhang, Z. Abliz and X. Zhang, *Anal. Chem.*, 2018, **90**, 9897–9903.

50 N. Abbassi-Ghadi, S. S. Antonowicz, J. S. McKenzie, S. Kumar, J. Huang, E. A. Jones, N. Strittmatter, G. Petts, H.

Kudo, S. Court, J. M. Hoare, K. Veselkov, R. Goldin, Z. Takáts and G. B. Hanna, *Cancer Res.*, 2020, **80**, 2764–2774.

51 S. Ishikawa, I. Tateya, T. Hayasaka, N. Masaki, Y. Takizawa, S. Ohno, T. Kojima, Y. Kitani, M. Kitamura, S. Hirano, M. Setou and J. Ito, *PLoS One*, 2012, **7**, e48873.

52 Z. Liu, Z. Zhang, H. Mei, J. Mao and X. Zhou, *Hepatol. Int.*, 2020, **14**, 544–555.

53 E. B. Evangelista, S. A. Kwee, M. M. Sato, L. Wang, C. Rettenmeier, G. Xie, W. Jia and L. L. Wong, *Diagnostics 2019, Vol. 9, Page 167*, 2019, **9**, 167.

54 K. Saito, E. Arai, K. Maekawa, M. Ishikawa, H. Fujimoto, R. Taguchi, K. Matsumoto, Y. Kanai and Y. Saito, *Sci. Rep.*, 2016, **6**, 28932.
